# Vegan Diets and Hypothyroidism

**DOI:** 10.3390/nu5114642

**Published:** 2013-11-20

**Authors:** Serena Tonstad, Edward Nathan, Keiji Oda, Gary Fraser

**Affiliations:** 1 Department of Health Promotion and Education, School of Public Health, Loma Linda University, Loma Linda, CA 92350, USA; 2 Department of Endocrinology, Morbid Obesity and Preventive Medicine, Section of Preventive Cardiology, Oslo University Hospital, Oslo N-0407, Norway; 3 Department of Health Promotion and Education, School of Public Health, Loma Linda University, Loma Linda, CA 92350, USA; E-Mail: enathan@llu.edu; 4 Department of Epidemiology and Biostatistics, School of Public Health, Loma Linda University, Loma Linda, CA 92350, USA; E-Mail: koda@llu.edu; 5 Department of Cardiology, School of Medicine, Loma Linda University, Loma Linda, CA 92350, USA; E-Mail: gfraser@llu.edu; 6 Department of Epidemiology and Biostatistics, School of Public Health, Loma Linda University, Loma Linda, CA 92350, USA

**Keywords:** vegan, hypothyroidism, diet, prevalence, incidence

## Abstract

Diets eliminating animal products have rarely been associated with hypothyroidism but may protect against autoimmune disease. Thus, we investigated whether risk of hypothyroidism was associated with vegetarian compared to omnivorous dietary patterns. The Adventist Health Study-2 was conducted among church members in North America who provided data in a self-administered questionnaire. Hypothyroidism was queried at baseline in 2002 and at follow-up to 2008. Diet was examined as a determinant of prevalent (*n* = 4237 of 65,981 [6.4%]) and incident cases (1184 of 41,212 [2.9%]) in multivariate logistic regression models, controlled for demographics and salt use. In the prevalence study, in addition to demographic characterstics, overweight and obesity increased the odds (OR 1.32, 95% CI: 1.22–1.42 and 1.78, 95% CI: 1.64–1.93, respectively). Vegan *versus* omnivorous diets tended to be associated with reduced risk (OR 0.89, 95% CI: 0.78–1.01, not statistically significant) while a lacto-ovo diet was associated with increased risk (OR 1.09, 95% CI: 1.01–1.18). In the incidence study, female gender, white ethnicity, higher education and BMI were predictors of hypothyroidism. Following a vegan diet tended to be protective (OR 0.78, 95% CI: 0.59–1.03, not statistically significant). In conclusion, a vegan diet tended to be associated with lower, not higher, risk of hypothyroid disease.

## 1. Introduction

There is a growing awareness that a plant-based diet decreases morbidity and mortality associated with a range of chronic disease. However, there are concerns that vegetarian diets may be low in calcium, vitamin D, vitamin B12, and zinc [1]. Another critical nutrient is iodine which is found mainly in iodized salt, breads and dairy sources in the U.S. Since 2000 the U.S. population generally has demonstrated adequate iodine nutriture [2]. Vegetarians may consume less iodine than their omnivorous counterparts [3,4]. The American Dietetic Association recommends that those consuming vegetarian diets be cognizant of the need of iodine supplementation [1].

Vegetarians include vegans who do not consume animal meat or dairy products, lacto-ovo vegetarians who eat eggs and dairy products and pesco-vegetarians who consume fish in addition to eggs and dairy. Lacto-ovo vegetarians in the U.K. appear to have adequate iodine intake [5]. Vegans may be at risk of inadequate iodine intake as animal products tend to be rich in iodine [4,6]. Study of British, and recently, U.S. vegetarians indicated that vegans are at risk of iodine deficiency [7,8]. Urinary iodine concentrations in Boston-area vegans were about one-half those observed in less strict vegetarians [8]. Transient neonatal hypothyroidism has been attributed to a maternal vegan diet [9].

The most common cause of acquired hypothyroidism is autoimmune thyroiditis [10]. In almost all cases, anti-thyroid antibodies are identified. The incidence is increased in women, with increasing age and is less common in Blacks. While hypothyroidism may cause obesity, obesity may result in raised thyrotropin-stimulating hormone levels, partly due to a proinflammatory milieu and other endocrine derangements [11]. Vegetarians tend to have lower body mass index (BMI) than nonvegetarians [12,13] and in some studies, lower levels of inflammatory markers [14]. Experimental data indicate that vegetarian diets may be useful in treatment of autoimmune disease as rheumatoid arthritis [15].

The purpose of this study was to see if vegetarian diets including vegan, lacto-ovo, pesco or semi-vegetarian were associated with prevalence and incidence of hypothyroidism compared to omnivore diets. The study was conducted in a church-going Adventist population exhibiting a wide range of dietary patterns from vegan to non-vegetarian.

## 2. Methods

The Adventist Health Study-2 (AHS-2) is a longitudinal study initiated to investigate the role of foods and their relationship to disease, particularly cancer. Participants were members of the Seventh-day Adventist church recruited through their respective churches in the U.S. and Canada. About 97,000 individuals joined between 2002 and 2006 [16]. Participants were eligible if they were proficient in English and were aged ≥30 years. All instruments and procedures were approved by the Loma Linda University Institutional Review Board in June 2001; approval was renewed annually thereafter.

### 2.1. Procedures

Once enrolled, each participant received a previously validated questionnaire with the informed consent materials [17]. The questionnaire was divided into sections on demographics, medical history, dietary patterns, physical activity and other lifestyle habits and self-reported height and weight. Questionnaires were returned by pre-paid envelopes and edited for missing data and stray marks. If the research staff felt data entries required confirmation, or demographic data were missing, a phone call was made to verify these entries.

Race and ethnicity were categorized as Black/African American, West Indian/Caribbean, African, other Black, Hispanic, and non-Hispanic White. In the current study all Blacks were collapsed into one category and non-Blacks in a second category. Respondents reported their level of education as grade school, some high school, high school diploma, some college, associate’s degree, bachelor’s degree, master’s degree, and doctoral degree. These data were collapsed into three categories: high school diploma or less, some college and college graduation or higher. Respondents reported personal and household income by checking one of eight income categories, ranging from <$10,000 to >$200,000. Responses were grouped into four categories: ≤$10,000, $11,000–20,000, $21,000–30,000 and >$30,000. BMI was calculated at baseline from respondents’ self-reported height and weight as kg/m^2^. These self-reports were validated [18].

### 2.2. Dietary Assessments

The food frequency questionnaire administered at baseline included over 200 items queried over the past year. Categorizing vegetarian status was done by defining vegans as subjects that reported consuming no animal products (red meat, poultry, fish, eggs, milk and dairy products < 1 time/month), lacto-ovo vegetarians as those who consumed dairy products and/or eggs ≥ 1 time/month but no fish or meat (red meat, poultry and fish < 1 time/month), pesco vegetarians as those who consumed fish ≥ 1 time/month and dairy products and/or eggs but no red meat or poultry (red meat and poultry < 1 time/month), semi-vegetarians as those who consumed dairy products and/or eggs and (red meat and poultry ≥ 1 time/month and <1 time/week) and omnivores as those who consumed animal products (red meat, poultry, fish, eggs, milk and dairy products > 1 time/week). The frequency of adding salt to food was queried as once a week or less, 2 to 6 times per week, and once per day or more. Iodine intake was not specified in the questionnaire.

To validate nutrient intakes, a calibration study was conducted among participants randomly selected from the AHS-2 cohort [19]. In this study validity coefficients were moderate to high for macronutrients, fatty acids, vitamin, mineral and fiber.

### 2.3. Outcome Assessments

In the medical history section of the questionnaire, respondents were asked if they had hypothyroidism diagnosed by a physician and if affirmative, to indicate when they were first diagnosed. Those who had been treated for hypothyroidism within the last 12 months were included as prevalent cases.

For assessment of incident disease, participants completed bi-annual Hospitalization History Questionnaires (HHQs) initially administered two years after the baseline questionnaire. In HHQ 3 administered in 2008, subjects were asked for the first time whether they had been diagnosed with hypothyroidism with the options of 2002–2004, 2005–2006 or 2007–2008.

### 2.4. Telephone Validation of Cases

A random sample of 103 cases reporting hypothyroidism in the prevalence study was generated. Up to three telephone calls were attempted to verify the diagnosis by asking the respondent to indicate whether they were treated for hypothyroidism at the baseline questionnaire. All 46 individuals that could be reached confirmed the diagnosis. Of the remaining, 33 did not reply to messages or subsequent calls, 16 had telephones that were disconnected or wrong numbers, and 8 hung up before answering the question.

### 2.5. Statistical Methods

Descriptive characteristics were compared between prevalent and incident cases and the rest of the population without hypothyroidism using chi-square or the two-sample *t*-test, as appropriate. Logistic regression was used to examine factors associated with the outcome (prevalence of hypothyroidism). The logistic regression model included gender, ethnicity, age (as continuous variable), BMI categorized as <25, 25–29.9 and ≥30 kg/m^2^, income, education, frequency of salt use and diet.

The denominator for incident cases was the population without prevalent disease who responded to HHQ 3 (*n* = 40,910). For incidence of hypothyroidism, we computed a complementary log-log model for interval-censoring in order to investigate the relationship between time-to-event and aforementioned risk factors [20]. The model also included the period of diagnosis as a covariate in the model. Results were presented as odds ratio (for the logistic model) and 95% confidence intervals. All analyses were done in SAS version 9.3 [21].

## 3. Results

Of about 97,000 respondents, complete data for all variables at baseline was available for 65,981. As shown in Table 1, several sociodemographic characteristics differed between subjects who were diagnosed with hypothyroidism at baseline (prevalence data) or reported new onset of hypothyroidism during follow-up. Subjects with hypothyroidism (prevalence and incidence data) were older, more likely to be female, less likely to be Black and more likely to use salt. In the prevalence data, these subjects had a higher BMI and lower income and education compared to subjects without hypothyroidism. Overall, diet differed between subjects with and without hypothyroidism in both datasets.

**Table 1:** Sociodemographic characteristics and diet according to diagnosis of hypothyroidism in the prevalence and incidence studies.

Variable	Prevalent Hypothyroidism *		Incident Hypothyroidism *	
	No	Yes	No	Yes
*N* (percentage)	61,744 (93.6)	4237 (6.4)	40,028 (97.8)	882 (2.2)
Age, mean (years)	56.3	62.6	56.6	58.8
BMI, mean (kg/m^2^)	27.2	28.2	26.8	26.9
Gender (percentage)				
Female	61.6	87.3	59.4	76.6
Race (percentage)				
Non-Black	74.6	93.5	81.7	94.2
Income (percentage)				
≤$10,000	19.4	26.5	17.8	22.6
$11,000–20,000	19.5	24.6	18.1	19.6
$21,000–30,000	16.8	16.1	16.4	16.4
>$30,000	44.3	32.7	47.7	41.4
Education (percentage)				
≤High school	18.7	18.1	16.6	14.2
Some college	39.3	44.8	37.4	39.0
≥College	42.0	37.2	46.0	46.8
Salt use (percentage)				
≤1 time/week	35.2	29.4	33.4	25.9
2–6 time/week	43.0	44.8	44.3	49.4
≥1 time/day	21.8	25.7	22.3	24.7
Diet (percentage)				
Vegan	8.3	7.0	8.9	7.0
Lacto-ovo	27.5	33.0	30.7	36.7
Semi	5.5	6.5	5.8	5.6
Pesco	9.8	8.8	9.4	7.6
Omnivore	48.9	44.7	45.2	43.1

* All variables differed between groups for prevalence (*p*-values < 0.0001). For incidence, *p*-values were <0.0001 for age, gender, race and salt use. For BMI, *p* = 0.76; for income, *p* = 0.0002; for education, *p* = 0.15 and for diet, *p* = 0.0015.

Table 2 shows that in multivariate logistic regression analysis, prevalent cases of hypothyroidism were associated with a lacto-ovo vegetarian diet, while a vegan diet tended to be protective.

**Table 2:** Odds ratios and 95% confidence intervals estimated by logistic multivariate analysis for prevalence of hypothyroidism (*N* = 65,981). Odds ratios are adjusted for all of the variables shown.

Odds Ratio	95% Confidence Interval	
Age (years)	1.03	1.03–1.04
Gender		
Women	Referent	
Men	0.21	0.19–0.23
Race		
Non-Black	Referent	
Black	0.21	0.19–0.24
BMI, kg/m^2^		
<25	Referent	
25–29.9	1.32	1.22–1.42
≥30	1.78	1.64–1.93
Income		
≤$10,000	Referent	
$11,000–20,000	0.98	0.89–1.07
$21,000–30,000	0.94	0.85–1.04
>$30,000	1.00	0.92–1.10
Education		
≤High school	Referent	
Some college	1.36	1.24–1.49
≥College	1.38	1.25–1.52
Salt use		
≤1 time/week	Referent	
2–6 time/week	1.11	1.03–1.20
≥1 time/day	1.15	1.05–1.25
Diet		
Vegan	0.89	0.78–1.01
Lacto-ovo	1.09	1.01–1.18
Semi	1.04	0.91–1.19
Pesco	1.02	0.90–1.15
Omnivore	Referent	

Table 3 shows that new cases of hypothyroidism tended to be reduced in vegan compared to omnivore subjects.

**Table 3:** Odds ratios and 95% confidence intervals estimated by the complementary log-log model for incidence of hypothyroidism (*N* = 40,910). Odds ratios are adjusted for all of the variables shown.

Odds Ratio	95% Confidence Interval	
Age (years)	1.01	1.01–1.02
BMI, kg/m^2^		
<25	Referent	
25–29.9	1.03	0.88–1.21
≥30	1.24	1.05–1.48
Gender		
Women	Referent	
Men	0.41	0.35–0.49
Race		
Non-Black	Referent	
Black	0.27	0.20–0.36
Income		
≤$10,000	Referent	
$11,000–20,000	0.93	0.76–1.14
$21,000–30,000	0.96	0.77–1.19
>$30,000	0.97	0.80–1.17
Education		
≤High school	Referent	
≤High school	1.27	1.03–1.57
≥College	1.45	1.17–1.80
Salt use		
≤1 time/week	Referent	
2–6 time/week	1.27	1.08–1.50
≥1 time/day	1.18	0.98–1.42
Diet		
Vegan	0.78	0.59–1.03
Lacto-ovo	1.07	0.91–1.24
Semi	0.87	0.65–1.17
Pesco	0.87	0.67–1.14
Omnivore	Referent	
Period of diagnosis		
2002–2004	Referent	
2005–2006	0.86	0.71–1.03
2007–2008	1.56	1.32–1.83

## 4. Discussion

Our main finding was that following a vegan diet tended to be associated with protection against hypothyroidism in the incidence and prevalence studies, though statistical significance was not attained. While vegan diets are associated with lower body weight, which may protect against hypothyroidism, the lower risk among vegans existed even after controlling for BMI and potential demographic confounders. Following a lacto-ovo vegetarian diet was associated with increased prevalent hypothyroidism but not incident hypothyroidism.

In regard to the tendency toward a lower risk of hypothyroidism associated with the vegan diet, the prevalence and incidence data were congruent, though not statistically significant. Several explanations may be given for our findings. As the diagnosis was not verified by objective measures of thyroid function, bias may exist. Vegans may be less likely to visit their physician for physical examinations than other dietary groups and thus disease may be underdiagnosed. We are unaware of published data to confirm this notion. Unmeasured confounding may be present, though we controlled for known confounders of the relation between diet and thyroid disease, including the lower BMI of vegans and a range of demographic variables. A postulated biological explanation is that animal products may induce an inflammatory milieu. Red meat consumption is associated with higher levels of high-sensitivity C-reactive protein (CRP) [22] and in the population studied herein we earlier found that following a vegetarian diet was associated with lower CRP levels [14]. However, this is solely a postulate, and little is known about autoimmune effects of vegetarian diets. Very sparse data is available on effects of vegetarian diets on rheumatoid arthritis and other autoimmune diseases [23].

We do not have a ready explanation for the findings related to the lacto-ovo dietary group, though several components of the vegetarian diet have been associated with increased risk of thyroid disease including soy products, cruciferous vegetables and low iodine intakes [2,3,4]. However, if these foods were causative of impaired thyroid function, one may expect the vegan group to carry as high risk as the lacto-ovo vegetarian group. Very early experiments in animals indicated concerns about goitrogenic effects of unheated soy [24]. There is also epidemiological evidence in infants to bolster this concept [25]. Among vegetarians with subclinical hypothyroidism, soy phytoestrogens may impair thyroid status [26]. On the other hand, most human clinical data do not show a significant causative effect of soy on hypothyroidism [27,28]. Goitergenic foods include cruciferous vegetables which suppress thyroid function, however, little evidence exists that these effects are clinically significant in the U.S. population.

Prevalent cases constituted ~6% of the population, which is somewhat higher than national estimates, probably due to the older age of the church going sample in this study. In comparison, NHANES III showed a prevalence of hypothyroidism of 4.6% [29]. Incident cases were calculated to be 0.36% per year, if incidence was equally distributed over the entire follow-up period of 6 years—however, this was not the case as shown in Table 3.

We found the expected associations between demographic characteristics and hypothyroidism in the current study. Studies universally demonstrate a higher incidence and prevalence of thyroid disease in women and non-Blacks [29]. Income was not associated with prevalent or incident disease. Yet, we found a relation between longer education and prevalent and incident cases. We are unaware of previous publications that found this relationship. This may imply under-diagnosis in the groups with less education.

The relation between obesity and hypothyroidism appears to have several explanations. Persons with obesity are prone to develop autoimmune hypothyroidism, and even mild thyroid failure contributes to the progressive increase in body weight, which ultimately results in overt obesity [30]. Furthermore, obese patients exhibit elevated thyrotropin-stimulating hormone levels, which may be the consequence, rather than the cause of obesity [11].

### Strengths and Limitations

The strengths of our study include the sizable cohort, following a range of diets and the availability of both cross-sectional and prospective data. While the dietary questionnaires were validated, iodine intake was not measured, so we used added salt as a proxy measure. However, the questionnaire did not specify whether it was iodized. We do not have data indicating whether vegans were more or less likely to use iodized salt than other dietary groups. Increased salt use was associated with increased hypothyroidism, both in the prevalence and incidence studies—this finding is not explained. We did not record the family history of thyroid disease which plays a part in pathogenesis of autoimmune thyroid disease.

The data are self-reported, and health risk behaviors and disease states may be under or over reported. However, under-reporting of events is unlikely to differ between self-administered questionnaires and blood samples [31]. Self-reported questionnaires suggest high sensitivity for hypothyroidism [32]. Individuals may report thyroid problems because of other medical symptoms, like weight loss. However, only participants who reported treatment in the last 12 months were considered to have the disease, to reduce over-reporting.

## 5. Conclusions

With the exception of the lacto-ovo vegetarian diet findings in the prevalence study, vegetarian diets were not associated with increased risk of hypothyroidism. Vegan diets which may be expected to lack iodine due to complete exclusion of animal products tended to be protective.

## References

[1] Craig W.J., Mangels A.R. (2009). Position of the American dietetic association: Vegetarian diets. J. Am. Diet. Assoc..

[2] Caldwell K.L., Makhmudov A., Ely E., Jones R.L., Wang R.Y. (2011). Iodine status of the U.S. population, National Health and Nutrition Examination Survey, 2005–2006 and 2007–2008. Thyroid.

[3] Krajcovicova-Kudlackova M., Buckova K., Klimes I., Sebokova E. (2003). Iodine deficiency in vegetarians and vegans. Ann. Nutr. Metab..

[4] Remer T., Neubert A., Manz F. (1999). Increased risk of iodine deficiency with vegetarian nutrition. Br. J. Nutr..

[5] Phillips F. (2005). Vegetarian nutrition. Nutr. Bull..

[6] Lightowler H.J., Davies G.J. (1998). Iodine intake and iodine deficiency in vegans as assessed by the duplicate-portion technique and urinary iodine excretion. Br. J. Nutr..

[7] Appleby P.N., Thorogood M., Mann J.I., Key T.J. (1999). The oxford vegetarian study: An overview. Am. J. Clin. Nutr..

[8] Leung A.M., LaMar A., He X., Braverman L.E., Pearce E.N. (2011). Iodine status and thyroid function of Boston-area vegetarians and vegans. J. Clin. Endocrinol. Metab..

[9] Shaikh M.G., Anderson J.M., Hall S.K., Jackson M.A. (2003). Transient neonatal hypothyroidism due to a maternal vegan diet. J. Pediatr. Endocrinol. Metab..

[10] Roberts C.G.P., Ladenson P.W. (2004). Hypothyroidism. Lancet.

[11] Rotondi M., Magri F., Chiovato L. (2011). Thyroid and obesity: Not a one-way interaction. J. Clin. Endocrinol. Metab..

[12] Tonstad S., Stewart K., Oda K., Batech M., Herring R.P., Fraser G.E. (2013). Vegetarian diets and incidence of diabetes in the Adventist Health Study-2. Nutr. Metab. Cardiovasc. Dis..

[13] Berkow S.E., Barnard N. (2006). Vegetarian diets and weight status. Nutr. Rev..

[14] Paalani M., Lee J.W., Haddad E., Tonstad S. (2011). Determinants of inflammatory markers in a Bi-ethnic population. Ethn. Dis..

[15] Kjeldsen-Kragh J., Haugen M., Borchgrevink C.F., Laerum E., Eek M., Mowinkel P., Hovi K., Førre O. (1991). Controlled trial of fasting and one-year vegetarian diet in rheumatoid arthritis. Lancet.

[16] Butler T., Fraser G.E., Beeson W.L., Knutsen S.F., Herring R.P., Chan J., Jaceldo-Siegl K. (2008). Cohort profile: The Adventist Health Study-2 (AHS-2). Int. J. Epidemiol..

[17] Knutsen S.F., Fraser G.E., Linsted K.D., Beeson W.L., Shavlik D.J. (2001). Comparing biological measurements of vitamin C, folate, α-tocopherol and carotene with 24-h dietary recall information in nonhispanic blacks and whites. Ann. Epidemiol..

[18] Bes-Rastrollo M., Sabate J., Jaceldo-Siegl K., Fraser G.E. (2011). Validation of self-reported anthropometrics in the Adventist Health Study 2. BMC Public Health.

[19] Jaceldo-Siegl K., Knutsen S.F., Sabate J., Beeson W.L., Chan J., Herring R.P., Fraser G.E. (2010). Validation of nutrient intake using an FFQ and repeated 24 h recalls in black and white subjects of the Adventist Health Study-2 (AHS-2). Public Health Nutr..

[20] Allison P.D. (2010). Survival Analysis Using SAS: A Practical Guide.

[21] (2012). SAS Software.

[22] Montonen J., Boeing H., Fritsche A., Schleicher E., Joost H.G., Schulze M.B., Steffen A., Pischon T. (2013). Consumption of red meat and whole-grain bread in relation to biomarkers of obesity, inflammation, glucose metabolism and oxidative stress. Eur. J. Nutr..

[23] Hagen K.B., Byfuglien M.G., Falzon L., Olsen S.U., Smedslund G. (2009). Dietary interventions for rheumatoid arthritis. Cochrane Database Syst. Rev..

[24] McCarrison R. (1933). A paper on food and goitre. Br. Med. J..

[25] Fort P., Moses N., Fasano M., Goldberg T., Lifshitz F. (1990). Breast and soy-formula feedings in early infancy and the prevalence of autoimmune thyroid disease in children. J. Am. Coll. Nutr..

[26] Sathyapalan T., Manuchehri A.M., Thatcher N.J., Rigby A.S., Chapman T., Kilpatrick E.S., Atkin S.L. (2011). The effect of soy phytoestrogen supplementation on thyroid status and cardiovascular risk markers in patients with subclinical hypothyroidism: A randomized, double-blind, crossover study. J. Clin. Endocrinol. Metab..

[27] Dillingham B.L., McVeigh B.L., Lampe J.W., Duncan A.M. (2007). Soy protein isolates of varied isoflavone content do not influence serum thyroid hormones in healthy young men. Thyroid.

[28] Messina M., Redmond G. (2006). Effects of soy protein and soybean isoflavones on thyroid function in healthy adults and hypothyroid patients: A review of the relevant literature. Thyroid.

[29] Hollowell J.G., Staehling N.W., Flanders W.D., Hannon W.H., Gunter E.W., Spencer C.A., Braverman L.E. (2002). Serum TSH, T(4), and thyroid antibodies in the United States population (1988 to 1994): National Health and Nutrition Examination Survey (NHANES III). J. Clin. Endocrinol. Metab..

[30] Michalaki M.A., Vagenakis A.G., Leonardou A.S., Argentou M.N., Habeos I.G., Makri M.G., Psyrogiannis A.I., Kalfarentzos F.E., Kyriazopoulou V.E. (2006). Thyroid function in humans with morbid obesity. Thyroid.

[31] Boekholdt S.M., Titan S.M., Wiersinga W.M., Chatterjee K., Basart D.C., Luben R., Wareham N.J., Khaw K.T. (2010). Initial thyroid status and cardiovascular risk factors: The EPIC-Norfolk prospective population study. Clin. Endocrinol..

[32] Brix T.H., Kyvik K.O., Hegedus L. (2001). Validity of self-reported hyperthyroidism and hypothyroidism: Comparison of self-reported questionnaire data with medical record review. Thyroid.

